# Health promotion intervention in mental health care: design and baseline findings of a cluster preference randomized controlled trial

**DOI:** 10.1186/1471-2458-12-431

**Published:** 2012-06-13

**Authors:** Nick Verhaeghe, Jan De Maeseneer, Lea Maes, Cornelis Van Heeringen, Veerle Bogaert, Els Clays, Dirk De Bacquer, Lieven Annemans

**Affiliations:** 1 Department of Public Health, Faculty of Medicine and Health Sciences, Ghent University, De Pintelaan 185, B-9000, Ghent, Belgium; 2 Department of Family Medicine and Primary Health Care, Faculty of Medicine and Health Sciences, Ghent University, De Pintelaan 185, B-9000, Ghent, Belgium; 3 Department of Psychiatry and Medical Psychology, Faculty of Medicine and Health Sciences, Ghent University, De Pintelaan 185, B-9000, Ghent, Belgium; 4 Department of Medical Sociology, Faculty of Medicine and Pharmacy, Vrije Universiteit Brussel, Laarbeeklaan 103, B-1090, Brussels, Belgium

**Keywords:** Health promotion, Intervention study, Physical activity, Eating habits, Mental health care

## Abstract

**Background:**

Growing attention is given to the effects of health promotion programs targeting physical activity and healthy eating in individuals with mental disorders. The design of evaluation studies of public health interventions poses several problems and the current literature appears to provide only limited evidence on the effectiveness of such programs. The aim of the study is to examine the effectiveness and cost-effectiveness of a health promotion intervention targeting physical activity and healthy eating in individuals with mental disorders living in sheltered housing. In this paper, the design of the study and baseline findings are described.

**Methods/design:**

The design consists of a cluster preference randomized controlled trial. All sheltered housing organisations in the Flanders region (Belgium) were asked if they were interested to participate in the study and if they were having a preference to serve as intervention or control group. Those without a preference were randomly assigned to the intervention or control group. Individuals in the intervention group receive a 10-week health promotion intervention above their treatment as usual. Outcome assessments occur at baseline, at 10 and at 36 weeks. The primary outcomes include body weight, Body Mass Index, waist circumference, and fat mass. Secondary outcomes consist of physical activity levels, eating habits, health-related quality of life and psychiatric symptom severity. Cost-effectiveness of the intervention will be examined by calculating the Cost-Effectiveness ratio and through economic modeling.Twenty-five sheltered housing organisations agreed to participate. On the individual level 324 patients were willing to participate, including 225 individuals in the intervention group and 99 individuals in the control group. At baseline, no statistical significant differences between the two groups were found for the primary outcome variables.

**Discussion:**

This is the first trial evaluating both the effectiveness and cost-effectiveness of a health promotion intervention targeting physical activity and healthy eating in mental health care using a cluster preference randomized controlled design. The baseline characteristics already demonstrate the unhealthy condition of the study population.

**Trial registration:**

This study is registered at clinicaltrials.gov – NCT 01336946

## Background

People with mental disorders (MD) are at increased risk for overweight (Body Mass Index 25–29 kg/m²) and obesity (Body Mass Index >30 kg/m²) compared with the general population [1,2]. Beside the side effects of especially atypical antipsychotics on body weight [3], the higher prevalence of these conditions is associated with more sedentary lifestyles, which include less mild or strenuous forms of physical activity (PA) [4,5], and poorer dietary choices compared with the general population [6,7].

Growing attention is given to the effects of lifestyle interventions targeting PA and healthy eating in mental health care. The importance of health promotion in mental health care is acknowledged by the European Psychiatric Association declaring that maintaining a healthy body weight and shape by healthy eating and regular PA is a key component in order to reduce the risk of some important somatic diseases and to improve the overall health and well-being of patients [8]. However, the current literature on weight reduction interventions in mental health care appears to provide only limited evidence on the effectiveness of either psycho-educational programs or programs combining educational and exercise components [9].

Most attention of health economic research goes to health economic evaluations of medicines and technologies. Recently, more emphasis is given to health economic evaluations of preventive health care. In general populations, these kind of studies yield no conclusive evidence [10,11], which is probably explained by wide differences in program contents. In mental health care, the cost-effectiveness of health promotion interventions targeting PA and healthy eating has thus far not been investigated [12]. Consequently, the study of both the effectiveness and cost-effectiveness of health promotion interventions targeting PA and eating habits in mental health care is required to determine and compare the efficiency of these kinds of interventions.

The design of evaluation studies of public health interventions, like health promotion interventions, poses several problems and they require multiple, flexible, and community driven strategies [13].

In most clinical trials, participants are randomized as individuals to intervention or control groups. However, when individual randomization is not possible or desirable, groups of individuals can be randomized to intervention or control groups [14]. This kind of design is known as a cluster or group or community randomized trial [15]. According to the British Medical Research Council [16] a cluster randomized design has to be considered when the intervention is designed to be delivered to groups rather than to individuals. Cluster randomization may also be appropriate when there is a risk of contamination, i.e. when individuals randomized to the intervention group may influence others within the group [15].

Another concern in studies evaluating behavioral or psychosocial interventions is that the participants are typically informed about their experimental assignment soon after randomization. Being assigned to a non-preferred intervention condition could be disappointing, or even demoralizing and reduce participants’ interest to participate so that they may withdraw from the study [17,18]. An alternative design for the randomized controlled trial is the ‘patient preference design’, in which subjects are allowed to select the intervention assignment. Preference designs are useful when strong preferences among potential participants threaten either the ability to recruit an adequate sample size of representative participants or when such preferences threaten participants’ acceptance of treatment assignment, adherence, or retention in the trial [19].

This paper describes the design and baseline findings of a health promotion intervention targeting PA and healthy eating in people with MD living in sheltered housing, whereby the above mentioned design issues were accounted for. Our design is innovative in a way that preference occurred at the level of the sheltered housing organisation (SHO) and not on the level of the individual patients. The description of the study protocol is in agreement with the checklist of the CONSORT statement for cluster randomized trials [20].

## Methods and design

### Aim of the study and hypotheses

The aim of the study is to evaluate the effectiveness and cost-effectiveness of a health promotion intervention targeting PA and healthy eating in people with MD living in sheltered housing. We hypothesize that:

· Between baseline and the end of the intervention and after a 6-month follow up period, significant differences in the primary outcomes ‘body weight’,‘Body Mass Index’ (BMI), ‘waist circumference’ (WC), ‘fat mass’ between the intervention and control group will be identified;

· Between baseline and the end of the intervention and after a 6-month follow up period, significant differences in the secondary outcomes ‘quality of life’ (QOL), ‘PA levels’, ‘eating habits’, and ‘psychiatric symptom severity’ between the intervention and the control group will be identified;

· The health promotion intervention is cost-effective.

### Study design and setting

**Figure 1:**
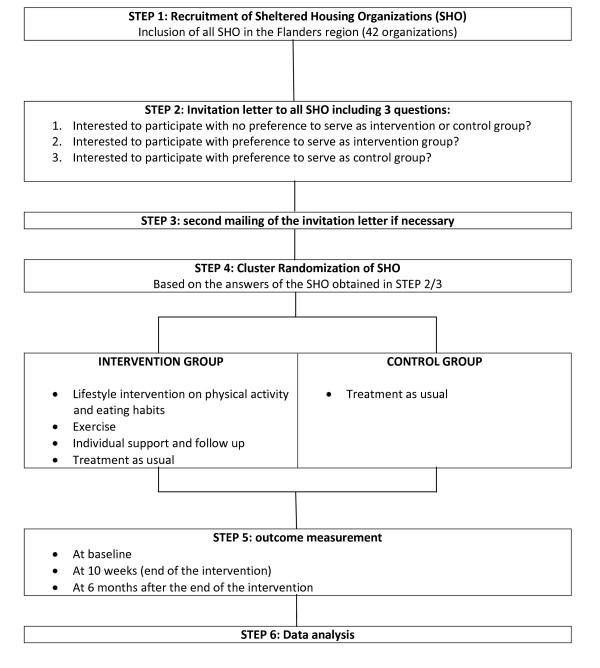
Health promotion intervention: Study design.

The design consisted of a cluster preference randomized controlled trial. An overview of the study design can be found in Figure 1. The study was conducted in sheltered housing organisations (SHOs) in the Flanders region (Belgium) with the SHOs as the unit of randomization. In SHOs, support on several domains (e.g. psychological, domestically, occupational, relational) is offered to the patients. In this type of health care service, patients are living alone in a studio or apartment or together with other patients in ‘community houses’. For this reason, cluster randomization was appropriate. Individual randomization at the level of the individual patient would decisively cause contamination bias due to the risk of participants in the intervention and control group living together. Cluster randomization to the intervention or control group therefore occurred at the level of the SHOs.

Mental health professionals working in the intervention SHOs were asked to lead and to support the health promotion intervention. This implied a significant involvement and workload above their usual workload. Also, as mentioned before, centers being assigned to a non-preferred intervention arm could be disappointed, which may reduce their interest to participate in the study [12,13]. Moreover, a substantial risk of non-participation was also assumed based on the results of previous qualitative research indicating that lack of time due to the high workload in the daily care of patients is a common barrier for mental health professionals to engage in health promotion programs [21,22]. Consequently, it appeared to be necessary to provide a detailed explanation about the expectations when serving as an intervention SHO. For this reason, preference randomization appears to be appropriate.

An invitation letter and response form with a self-addressed postage envelope was sent to the managers of all SHOs in the Flanders region. They were asked if they were interested to participate in the study having (i) no preference to serve as intervention or control group and to be randomized or, (ii) a preference to serve as intervention group (see a detailed description of the intervention below) or, (iii) a preference to serve as control group. A concise explanation of the aim of the study and of the expectations and content when participating as intervention group was included in the letter. If necessary, a second mailing was foreseen. If a SHO was not prepared to participate, they were asked to report the reason for non-participation.

Subsequently, based on the responses, SHOs were either assigned to the intervention or control group according to their preference or randomly assigned to the intervention or control group when they expressed no preference. Randomization occurred by an external person not involved in the study. Finally, the patients living in the intervention and control SHOs received both written and oral information about the study. The written information consisted of a detailed explanation about the study and an informed consent.

### Study population

The study population consisted of people with MD aged between 18 and 75 years living in a SHO in the Flanders region (Belgium). There are 42 SHOs in the Flanders region, including 2662 approved places [23]. Exclusion criteria included people aged <18 or older than 75 years, having a gastric ring or pacemaker placed, having cognitive impairments (assessed by the mental health professionals) compromising the understanding of the psycho-educational and behavioral sessions of the health promotion intervention.

### Development of the materials

**Figure 2:**
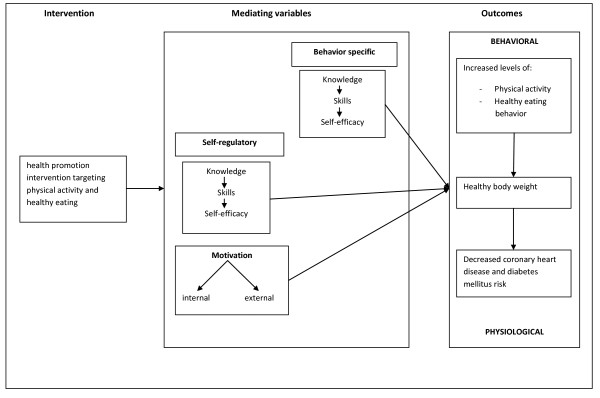
Theoretical framework of the health promotion intervention targeting physical activity and healthy eating.

The theoretical framework of the intervention was developed using elements of several theories including the social-cognitive theory [24], the self-determination theory [25], and the control theory [26]. The health promotion intervention was developed using the mediating variable approach including the mediating variables knowledge, skills, self-efficacy and motivation [24,25]. A schematic overview of the theoretical framework can be found in Figure 2. Knowledge is a necessary component of behavior change [24]. For example, how to select appropriate food portion sizes, how to distinguish between sedentary and moderate or vigorous physical activities. Behavior-specific skills are those specifically related to the targeted behavior [27]. For example, how to interpret the level of physical shape by measuring the pulse rate. Self-regulatory skills include goal setting and problem solving [27]. Self-efficacy is confidence in one’s ability to successfully perform a task or behavior and is influenced in two ways: personal success and observing others successfully perform the behavior [24]. Two types of motivation can be distinguished. People can be motivated because they value an activity either from a sense of personal commitment or because there is strong external motivation and support [25].

The staff manual was developed based on the manual ‘Health promotion on well-balanced eating and healthy physical activity’ developed by the Flemish Institute of Health Promotion and Disease Prevention [28]. As the target population of this manual is the general population, some adjustments were made to focus especially on the lifestyle behavior of our target population (for example how to choose a more healthy lifestyle despite the presence of barriers associated with the MD). The manual was built around ten themes focusing on PA and healthy eating: 1) PA and healthy eating: Introduction, 2) Awareness of the consumption of fat and fibres, 3) A healthy lifestyle: advantages and barriers, 4) The food triangle, 5) Using the food triangle throughout the day, 6) Label reading, 7) The influence of the environment & Budget issues, 8) & 9) Physical activity, 10) A quiz regarding PA and healthy eating.

### Study duration and intervention components

The study period consists of an intervention period of 10 weeks followed by a post-intervention period of six months. In addition to treatment as usual, the intervention groups (n = 14) receive the 10-week health promotion program targeting PA and healthy eating. In the intervention group the following intervention components are offered:

· Psycho-educational and behavioral group sessions

This part of the program consists of 10 group sessions in a 10-week period and includes discussions on PA and healthy eating, problem solving, written exercises, quizzes and plans to increase PA levels and to stimulate a more healthy eating behavior. All participants in the intervention group receive the same information in the same format. The program is delivered by the mental health professionals working in the intervention SHOs.

· Supervised exercise

In the same 10-weeks period a weekly 30-minutes supervised walking session is organized. These sessions are also led by one or more mental health professionals.

· Individual counseling

During the 10-week intervention period, all participants in the intervention group receive individual support from the mental health professionals (for example motivation to persist, discussing of experiences).

### Implementation of the intervention

The manager of each SHO in the intervention group was asked to discuss with their team of mental health professionals the selection of one or two persons, who would serve as contact person with the research team and who would be responsible for the sessions. Every intervention SHO was visited by the same researcher. The aim of this visit was to instruct the mental health professionals who would lead and supervise the sessions. Preferably, also other staff members were present during this training session. During the study period, it is possible to contact one of the researchers by phone or e-mail. If necessary, visits of one of the researchers to the SHO will also be possible.

### Evaluation of the intervention

At the end of the study period, a process evaluation of the health promotion program will be organized for all participating SHOs in the intervention group. This evaluation will consist of a questionnaire with both closed and open-ended questions including topics on experiences, advantages and disadvantages of the program, lessons learned, and suggestions for further research.

### Sample size calculation

The sample size calculation is based on an average change of the primary outcome body weight of 3.5 kg between the intervention and the control group at the end of the study. This change is based on the results of a previous literature review performed by the research team on the effectiveness and cost-effectiveness of lifestyle interventions on PA and eating habits in people with MD [12]. Cluster randomized trials require larger sample sizes than the individually randomized design. This can be explained by the fact that observations on individuals in the same cluster tend to be correlated, and so the effective sample size is less than the total number of individual participants. This reduction in effective sample size and the degree of correlation within clusters is known as the intraclass correlation coefficient (ICC) [20,29]. As no ICC for this kind of intervention in people with MD was found in the literature, an assumption was made by multiplying the sample size with a design factor of 1.5. A sample size of 371 participants in each group would provide a sample large enough to detect a difference in mean body weight change of 3.5 kg across the two groups with 80% power at a significance level of 0.05.

### Data collection and outcome measurements

#### Sociodemographics

Participants will be asked to complete a questionnaire on sociodemographics including sex, age, duration of stay in sheltered housing, marital status, occupational status, contacts with relatives, tobacco and alcohol use, and medication use.

#### Primary outcome measures

The primary outcomes of the study consists of changes in body weight, BMI, WC and fat mass. Body weight is measured in all participants wearing light clothing without shoes by a member of the research team using a TANITA BC-420 SMA digital weighing scale (TANITA, Tokyo, Japan). A member of the research team measures height in a standardized way using a Seca 225 stadiometer (Seca GmbH & KG, Hamburg, Germany). The BMI is calculated by dividing the body weight in kilograms by the square of the height in meters. WC is measured with a Seca 200 tape (Seca GmbH & KG, Hamburg, Germany) by one of the researchers according to the guidelines described in the National Heart, Lung, and Blood Institute report ‘Clinical guidelines on the identification, evaluation, and treatment of overweight and obesity in adults. The evidence report’ [30]. The calculation of the fat mass occurs using the TANITA BC-420 SMA digital weighing scale (TANITA, Tokyo, Japan). This occurs at the same time as the weight assessment. Both the stadiometer and the digital weighing scale were placed on a flat surface to assure correct measurement of height, body weight, and fat mass.

#### Secondary outcome measures

Changes in PA are assessed using the Dutch long version of the self-administered International Physical Activity Questionnaire (IPAQ), as this questionnaire appears to be a reliable and valid PA measurement tool [31]. The analysis of the IPAQ is based on self-reported data. Therefore, PA levels are also measured using pedometers as a more objective tool. The Yamax Digiwalker SW-200 (Yamax, Tokyo, Japan) is used as this is known as accurate and reliable for counting steps [32]. Dietary habits of the participants are assessed using an adapted version for adults of the “Young Children’s Nutrition Assessment on the Web ”[33]. Quality of life is assessed using the SF-36 Health Survey questionnaire. Finally, psychiatric symptom severity is assessed through the use of the Brief Symptom Inventory (BSI). This questionnaire is considered as a reliable and a valid tool useful in patient groups with different psychiatric diagnoses [34].

Data on all primary and secondary outcome measures are collected at baseline and at 10 weeks. At the end of the study (at 36 weeks) only data on body weight, WC and fat mass will be collected and BMI will be calculated. At that time participants will be asked to only complete again the SF-36 Health Survey.

#### Cost-effectiveness

As there is growing need for health economic research in health care and health policy, the cost-effectiveness of the health promotion intervention will also be examined. This will occur by calculating the difference in costs between intervention and no intervention (usual care), by calculating the expected health gain expressed in quality-adjusted life years (QALY) through health economic modeling, and by calculating the Incremental Cost-Effectiveness Ratio (ICER). The ICER is calculated as the ratio of the net cost to the net health gain: ICER = (COST_I_ – COST_NI_)/(QALY_I_ –QALY_NI_) where I is intervention and NI is no intervention.

QALYs are calculated by multiplying the utility level for a given disease status (a health-related quality-of-life weight ranging between 0 and 1) with the number of years an individual suffers from that disease. A utility of 0 is assigned to death, while an utility of 1 represents perfect health.

### Data analysis

Parametric and non-parametric tests are used at the individual level to compare the intervention and control group at baseline, depending on the distributions of the quantitative variables. The X²-test is used in qualitative variables. Repeated measure analyses will be used to evaluate differences in the primary outcome variables body weight, BMI, WC and fat mass between pre- and post-intervention in the intervention and control group. Because preference randomization occurred at the level of SHO and not at the level of the individual patient, cluster effects will also be examined. The analyses of the primary outcomes will be performed on an intention to treat (ITT) basis. Secondary outcome variables will be evaluated per protocol. A P-value ≤0.05 is considered statistically significant. For statistical analyses, SPSS®19 will be used.

To examine the cost-effectiveness of the intervention, a Markov decision-analytic model assuming a public payer perspective will be constructed to project health outcomes and costs of the health promotion intervention compared with usual care. Overweight and obesity are substantial risk factors for the high prevalence of type 2 diabetes and cardiovascular disease in individuals with MD[35,36]. Therefore, the Markov model will be used to estimate, for both the intervention and control group, the development of cardiovascular disease and type 2 diabetes over time and the associated costs. The time horizon will be a 10-year period. In both the intervention and control arm several health states will be included in the Markov model. All future costs and health outcomes will be discounted respectively at 3% and 1.5% annually. The costs will be calculated by analyzing the direct health care costs and the costs of the program. The direct health care costs include hospitalization, medication, GP consultations, and other health professionals costs. The program related resource use (staff time, materials) and resulting costs will be calculated making a distinction between the resource used and costs related to the research purpose and those related to the intervention itself.

### Ethics

Permission to perform the study was obtained from the Ethics Committee of the University Hospital of Ghent (Belgium). Written consent for participation is obtained from all participants. Participation in the study is voluntary and all participants are informed that the data analysis will be anonymous and that they could withdraw from the study at any time. A reward (pedometer) for the participants in the intervention group who completed the program was foreseen.

## Baseline study population characteristics

**Figure 3:**
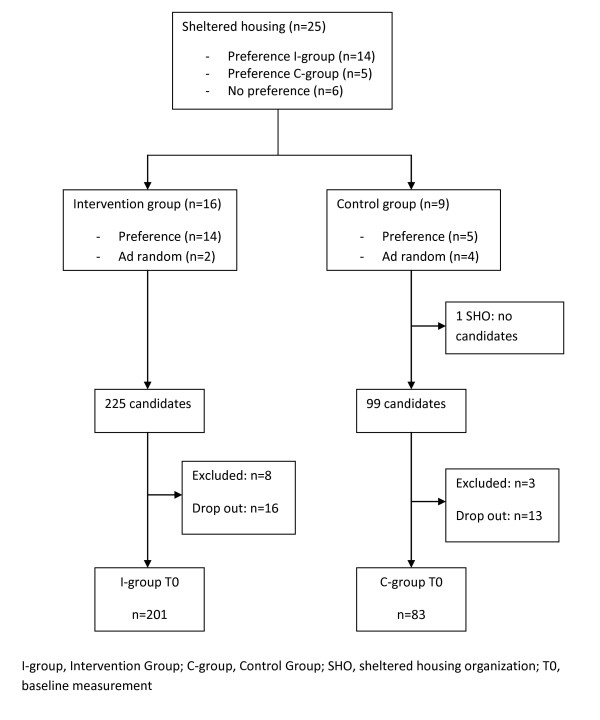
Health promotion intervention: Recruitment process.

A schematic overview of the recruitment process is shown in Figure 3. Twenty-five SHOs were interested to participate. Fourteen of these expressed a preference to serve as intervention group, while five preferred to serve as control group. Six expressed no preference neither for the intervention nor for the control group. These six were randomly assigned to the intervention group (n = 2) and to the control group (n = 4). In one of the SHOs serving as controls no patients were interested to participate.

On the individual level 324 patients were willing to participate, including 225 and 99 candidates in respectively the intervention and control SHOs. This accounted for a response rate of 24% in the intervention group and 21.1% in the control group. Eleven respondents were excluded because of age (n = 2), cognitive impairments (n = 4), having a gastric ring placed (n = 1), and the impossibility to be weighed using the digital weighing scale (n = 4). Of these four patients, three had a pacemaker and one patient had an artificial limb. Twenty-nine (9.3%) of the remaining 313 candidates withdrew before the baseline measurement due to hospital admission (n = 4), no further interest (n = 24), and one patient died in the period prior to the baseline measurement. This resulted in 284 patients for whom baseline data are available.

The sociodemographic characteristics for the overall study population and by treatment group are listed in Table 1. The overall study population consisted of 174 men (61.3%) and mean age at enrollment was 46.3 years. Two-thirds of the patients (67.7%) were living together. Sixty-one percent of the participants were daily smokers and 46% used alcohol on a regular basis. The most frequent psychiatric diagnosis consisted of schizophrenia (37.9%), followed by mood disorders (24.5%). Mean baseline body weight was 87.1 kg. Sixty point three percent of men had a baseline WC of >102 cm. In women, 86.4% had a WC of >88 cm. Of the total sample, 33.4% were classified as overweight and 47.2% as obese (Table 2).

**Table 1:** Baseline sociodemographic data

**Variable**	**All (n = 284)**	**Intervention group (n = 201)**	**Control group (n = 83)**	***p***
Sex				.27§
men, *n (%)*	174 (61.3)	119 (59.2)	55 (66.3)	
women, *n (%)*	110 (38.7)	82 (40.8)	28 (33.7)	
Age (years), *mean ± SD*	46.3 ± 12.3	46.2 ± 12.5	46.5 ± 11.9	.83‡
Smoking status, *n (%)*				.22§
no smoking	103 (39)	77 (41.4)	26 (33.3)	
smoking	161 (61)	109 (58.6)	52 (66.7)	
Alcohol use, *n (%)*				.81§
regular	122 (46)	87 (46.5)	35 (44.9)	
never	143 (54)	100 (53.5)	43 (55.1)	
Employment, *n (%)*				.51§
regular	13 (4.9)	11 (5.9)	2 (2.5)	
sheltered	105 (39.6)	73 (39.2)	32 (40.5)	
no	147 (55.5)	102 (54.8)	45 (57.0)	
Living situation, *n (%)*				<.05§
alone	86 (32.3)	71 (38)	15 (19)	
with others	180 (67.7)	116 (62)	64 (81)	
Stay in SH (years), median *(range)*	4 (0.1–22.3)	4.4 (0.1–22.3)	2.5 (0.1–16.3)	<.05†
DSM-IV diagnosis, *n (%)*				
schizophrenia	105 (37.9)	80 (41.2)	25 (30.1)	.08§
mood disorder	68 (24.5)	44 (22.7)	24 (28.9)	.27§
substance misuse	44 (15.9)	30 (15.5)	14 (16.9)	.77§
personality disorder	40 (14.4)	29 (14.9)	11 (13.3)	.71§
other	20 (7.2)	11 (5.7)	9 (10.8)	.13§
Medication, *n (%)*				
sedatives/anxiolytica	7 (2.8)	3 (1.7)	4 (5.7)	.09§
antipsychotics	56 (22.8)	46 (26.1)	10 (14.3)	.05§
antidepressants	12 (4.9)	8 (4.5)	4 (5.7)	.70§
sedatives/antipsych./antidepres.	49 (19.9)	31 (17.6)	18 (25.7)	.15§
sedatives/antipsychotics	20 (8.1)	16 (9.1)	4 (5.7)	.38§
sedatives/antidepressants	16 (6.5)	9 (5.1)	7 (10.0)	.16§
antipsychotics/antidepressants	75 (30.5)	58 (33.0)	17 (24.3)	.18§
no medication	11 (4.5)	5 (2.8)	6 (8.6)	

§Pearson Chi-Square.

‡Independent samples *t*-test.

†Mann–Whitney *U* test.

SH, sheltered housing; DSM-IV, Diagnostic and Statistical Manual of Mental Disorders.

**Table 2:** Baseline anthropometric data

**Variable**	**All (n = 284)**	**Intervention group (n = 201)**	**Control group (n = 83)**	***p***
Weight mean ± SD *(kg)*	87.1 ± 19.5	87.9 ± 20.7	85.2 ± 16.0	.23‡
BMI mean ± SD *(kg/m²)*	30.0 ± 5.9	30.2 ± 6.1	29.5 ± 5.4	.37‡
Waist circumference mean ± SD *(cm)*	105.9 ± 16.1	106.2 ± 16.8	105.2 ± 14.4	.65‡
men wc >102cm, *n (%)*	105 (60.3)	76 (63.9)	29 (52.7)	.16§
women wc >88 cm, n (%)	95 (86.4)	70 (85.4)	25 (89.3)	.60§
Fat mass mean ± SD *(%)*	33.9 ± 10.6	34.2 ± 10.5	33.4 ± 10.6	.57‡
BMI class, *n (%)*				.70§
underweight (<18.5)	3 (1.1)	3 (1.5)	0 (0)	
normal weight (18.5–24.9)	52 (18.3)	36 (17.9)	16 (19.3)	
overweight (25–29.9)	95 (33.4)	66 (32.8)	29 (34.9)	
obesity (≥30)	134 (47.2)	96 (47.8)	38 (45.8)	

§Pearson Chi-Square.

‡Independent samples *t*-test.

kg., kilogramme; BMI, Body Mass Index (kg/m²); WC, waist circumference.

No statistical significant difference between the intervention and the control group was found for the primary outcome variables ‘body weight’ (p = .23), BMI (p = .37), WC (p = .65) and fat mass (p = .57). No statistical significantly differences were observed between the intervention and control group for the sociodemographic variables, except for the variables ‘living situation’, and ‘duration of stay in sheltered housing’. Length of stay in sheltered housing was significantly longer in participants in the intervention group (median 4.4 vs. 2.5, p < .05). A statistically higher proportion of controls was living together compared with those in the intervention group (81 vs. 62%, p < .05). Psychotropic medication use was found to be statistically different between the two groups for only the antipsychotics (p = .05).

## Discussion

In this paper the study design and baseline characteristics of a health promotion intervention targeting PA and healthy eating in people with MD living in sheltered housing is described. At baseline, statistical analysis of the characteristics found only a significant difference between the intervention and the control group for the variables ‘living situation’, ‘duration of stay in sheltered housing’, and antipsychotic medication use.

The baseline characteristics demonstrate the unhealthy condition of the study population. A higher prevalence of overweight and obesity was found in the study population compared with the general population in Belgium. Eighty percent of the study population has a BMI of >25 of which 47% is classified as obese, compared with respectively 47 and 14% in the general population [37]. Smoking prevalence in the study population also exceeds that of the general population in Belgium. Amongst the study population, 61% are daily smokers compared with 21% in the general Belgian population [37]. According to the results of several studies, the measurement of the WC and waist-hip ratio is more appropriate than measuring the BMI to estimate the risk for future cardiovascular events [38,39]. A WC above 102 cm for men and 88 cm for women is associated with an increased risk of developing health problems such as cardiovascular disease, type 2 diabetes and hypertension [30]. Sixty percent of men and 86% of women in our study population had a baseline WC above this threshold.

Given the high burden of overweight and obesity in people with MD, research on the effectiveness and cost-effectiveness of lifestyle interventions in this population is of substantial importance. Yet, there are several challenges in setting up trials involving individuals with MD. Previous research has identified a number of barriers to engagement in health promotion programs like the MD itself, side effects of psychotropic medication, financial barriers, poor motivation or unwillingness to participate, and absence of support [40-42].

According to the results of previous research on the effectiveness of weight management interventions in mental health care, significant reductions in weight gain are possible [43,44]. The results of these trials must however be interpreted cautiously because they are frequently limited by small sample sizes and short intervention periods. It is nevertheless promising that small weight reduction in this population is possible.

As far as known to the authors, this is the first trial evaluating both the effectiveness and cost-effectiveness of a health promotion intervention targeting PA and healthy eating in mental health care. Cost-effectiveness evaluations have a great social value as health promotion and prevention have an economic cost, but they can also increase healthy life expectancy and save money because diseases and complications can be avoided.

It is likely that the results of this intervention in SHOs will lead to further health promotion programs targeting other populations in mental health care, such as inpatients.

## Abbreviations

BMI, Body Mass Index; BSI, Brief Symptom Inventory; WC, waist circumference; GP, general practitioner; I, intervention; ICC, intraclass correlation coefficient; ICER, incremental cost-effectiveness ratio; IPAQ, International Physical Activity Questionnaire; ITT, intention to treat; kg, kilogramme; MD, mental disorder; NI, no intervention; PA, physical activity; QALY, quality-adjusted life year; QOL, quality of life; SHO, sheltered housing organisation; vs., versus.

## Competing interests

The authors declare that they have no competing interest.

## Authors’ contributions

NV is the primary researcher of the health promotion intervention study and drafted the manuscript, DDB participated in the design, data analysis and interpretation of the data. EC participated in the data analysis and interpretation of the data. LM participated in the design of the health promotion intervention. VB participated in the data collection and data input. LA and LM supervise the study. All authors have been involved in revising the manuscript and approved the final manuscript.

## Pre-publication history

The pre-publication history for this paper can be accessed here:

http://www.biomedcentral.com/1471-2458/12/431/prepub
